# Preoperative Tyrosine Kinase Inhibitors Before Percutaneous Cryoablation for Clinical T1b Renal Tumors

**DOI:** 10.7759/cureus.60345

**Published:** 2024-05-15

**Authors:** Kosuke Iwatani, Shoji Kimura, Fumihiko Urabe, Akihiro Matsukawa, Koichi Aikawa, Takafumi Yanagisawa, Kanichiro Shimizu, Kenta Miki, Takahiro Kimura, Jun Miki

**Affiliations:** 1 Department of Urology, The Jikei University School of Medicine, Kashiwa Hospital, Chiba, JPN; 2 Department of Urology, The Jikei University School of Medicine, Tokyo, JPN; 3 Department of Radiology, The Jikei University School of Medicine, Kashiwa Hospital, Chiba, JPN

**Keywords:** tyrosine kinase inhibitor, ct1b, presurgical treatment, renal cell carcinoma, cryoablation

## Abstract

Purpose

To treat renal cell carcinoma, local ablative therapy is a viable alternative treatment option. Traditionally, cryoablation has been used for the treatment of T1a renal tumors. However, recent technological developments have expanded its application to encompass select T1b renal tumors. Here, we present a retrospective study of the utilization of preoperative tyrosine kinase inhibitors (TKIs) to induce tumor shrinkage and achieve favorable outcomes in percutaneous cryoablation (PCA).

Methods

We retrospectively evaluated the data from nine patients with clinical T1b renal tumors who underwent PCA. Six patients with TKI pretreatment at our institution between 2016 and 2018 were included in the study. We evaluated the safety and efficacy of preoperative TKIs prior to PCA.

Results

All patients received axitinib with a median treatment duration of 80.5 days (IQR: 49-85). All patients experienced tumor shrinkage (median: 13.5 mm; IQR: 7-16); five experienced downstaging to T1a following tumor shrinkage. There were no severe adverse events (common terminology criteria for adverse events (CTCAE) grade ≥ 3) in TKIs. After the discontinuation of TKIs for two weeks, all PCA procedures were performed successfully without any severe complications. During a median follow-up of 46 months, no local recurrence was observed in any of these cases.

Conclusion

In cases with large renal tumors, TKI pretreatment prior to PCA had potential benefits in terms of tumor shrinkage and long-term local control rate. Further well-designed studies in larger populations are needed to validate our findings.

## Introduction

Nephrectomy and nephron-sparing nephrectomy are the gold-standard treatments for renal cell carcinoma. However, advances in technology, such as robotic surgery, have enabled nephron-sparing interventions for larger tumors, including T1b and T2a lesions [1]. Nevertheless, managing older adults with coexisting comorbidities and unilateral kidney involvement presents significant challenges. Ablation therapy has emerged as a promising solution to address these issues.

Ablation therapy, such as radiofrequency ablation, percutaneous cryoablation (PCA), and high-intensity focused ultrasound (HIFU), aims to achieve local control of renal cell carcinoma. Recent studies have demonstrated the comparable efficacy of PCA and nephron-sparing surgery for T1b tumors [2]. However, the tumor location and size may make these treatments difficult due to the potential adverse effects on adjacent organs, blood vessels, and the urinary tract. There have been several reports on the efficacy of preoperative molecular-targeted drugs for T1b tumors in nephron-sparing surgery [3,4]. However, only one case report regarding local ablative therapy is available in the literature [5]. Here, we present six cases where the utilization of tyrosine kinase inhibitors (TKIs) prior to PCA resulted in tumor shrinkage and successful local control.

## Materials and methods

Methods

We retrospectively evaluated the records of nine patients with cT1b renal tumors treated with cryoablation in our institution between 2016 and 2018. We report six patients treated preoperatively with TKIs. The exclusion criteria for preoperative treatment were as follows: (1) tumors in a location where cryoablation would not adversely affect adjacent organs; (2) tumors that are exophytic and not in contact with the renal pelvis; (3) tumors for which cryoablation could technically be performed without presurgical procedures; and (4) patients who had difficulty using molecular-targeted drugs. In this retrospective study, we assessed the efficacy of TKIs on tumor reduction, adverse events/complications, and postoperative local control outcomes. Tumor size was calculated by maximal diameter using contrast-enhanced computed tomography (CT) in both pre- and post-TKI treatments. Adverse events during TKI administration were evaluated according to the Common Terminology Criteria for Adverse Events (CTCAE).

Surgical procedure

CT-guided PCA was performed under local anesthesia using the CryoHit (Galil Medical, Yokneam, Israel) equipment. The 17-gauge cryoneedles (IceRod, Galil Medical, Yokneam Israel) were precisely positioned under CT guidance. As part of the standard protocol, tumor lipiodol marking and transcatheter arterial embolization (TAE) utilizing iodized oil were routinely performed two to four days prior to PCA [6]. The cryoneedle placement was performed percutaneously, guided by the location of the iodized oil deposit observed on plain CT images. A total of 2-10 cryoneedles employed were determined based on tumor diameter, following the established protocol at our institution [7]. Postoperative follow-up involved performing contrast-enhanced CT scans every three months to check for recurrence. In cases where contrast administration was not feasible, MRI and ultrasound examinations were conducted to ensure the absence of recurrence.

## Results

Patient demographics

Table 1 presents a comprehensive summary of the six cases included in this study.

**Table 1:** Case summary This is a summary of the cases. The patients were at high risk for hemorrhage, and no prior biopsy was performed in two cases. All cases showed a shrinkage in tumor size. RCC: renal cell carcinoma; CTCAE: Common Terminology Criteria for Adverse Events; ccRCC: clear cell renal cell carcinoma.

Case	Gender	Age (Years)	Reasons for Surgical Infeasibility	Biopsy	RCC Type	Tumor Location	Tumor Size (mm)		R.E.N.A.L Score		Medication	Dose Usage (mg)	Duration (Days)	Adverse Event (CTCAE Grade)
							Pre	Post	Pre	Post				
1	Male	72	Solitary kidney, multiple tumors	Yes	ccRCC	Left/lower pole	53	33	10a	8	Axitinib	10	49	Hypertension (G1), hypothyroidism (G2)
2	Male	82	Complications	None	Unknown	Right/middle	41	27	10p	8p	Axitinib	6	98	Hypothyroidism (G2)
3	Male	68	Complications	None	Unknown	Right/lower pole	70	54	8	7	Axitinib Sunitinib	10 37.5	140	Dizziness (G1), proteinuria (G2)
4	Female	79	Solitary kidney, multiple tumors	Yes	ccRCC	Right/upper pole	41	29	10p	8p	Axitinib	6	76	Thrombocytopenia (G2), hypertension(G2), Hypothyroidism (G2)
5	Male	75	Bilateral, multiple tumors, complications	Yes	ccRCC	Right/lower pole	46	39	8p	7p	Axitinib	5	85	Hypertension (G2), hypothyroidism (G2)
6	Male	67	Solitary kidney, multiple tumors	Yes	ccRCC	Left/upper pole	42	38	6p	5p	Axitinib	10	43	Diarrhea (G1)

The median age was 73.5 years (IQR: 68-79), and the median tumor size at presentation was 44 mm (IQR: 41-53). Axitinib was administered in all cases; however, in one patient, axitinib was substituted with sunitinib due to severe proteinuria (CTCAE grade 2). The median duration of preoperative medication was 80.5 days (IQR: 49-85), with a two-week discontinuation period preceding PCA. There were no adverse events of grade 3 or higher; nonetheless, grade 2 hypothyroidism, thrombocytopenia, diarrhea, and dizziness were documented. Notably, all of these side effects were effectively resolved either through dose reduction or discontinuation of TKIs. Tumors reduced in size in all cases (median: 13.5 mm; IQR: 7-16).

All PCA procedures were performed successfully without any severe complications (Table 2). During a median follow-up of 46 months following PCA, no local recurrence was detected in any cases, indicating a local control rate of 100%. The detailed clinical courses of each case are presented below.

**Table 2:** Perioperative outcome All patients underwent lipiodol marking with preoperative transarterial embolization. There was no significant decrease in renal function in all patients; hematuria and pneumothorax were noted in two patients, both of which improved conservatively. TAE: transcatheter arterial embolization; PCA: percutaneous cryoablation; eGFR: estimated glomerular filtration rate; CTCAE: Common Terminology Criteria for Adverse Events.

Case	Contrast Agent CT	TAE	Renal Function (eGFR mL/min/1.73m^2^)			Number of Needles (n)	Complication (CTCAE Grade)	Follow-up (Months)
			Before TAE/PCA	After PCA	3 months after PCA			
1	None	Yes	11.9	11.2	12	3	None	46
2	Yes	Yes	28.9	24.4	23.4	4	Hematuria (G1)	8
3	Yes	Yes	54.6	45.9	55.1	4 (10 punctures)	Pneumothorax (G1)	59
4	Yes	Yes	38	23	31.7	4	None	47
5	Yes	Yes	30.6	26.2	30.7	4 (7 punctures)	None	15
6	Yes	Yes	49.6	46.5	46.1	4	None	60

Case presentations

Case 1

**Figure 1:**
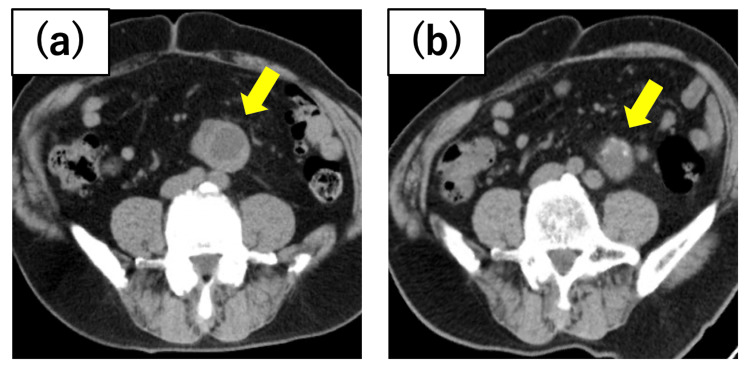
Case 1 renal tumor After a tumor biopsy diagnosed clear cell renal cell carcinoma (ccRCC) in the horseshoe kidney, preoperative axitinib administration resulted in the shrinkage of the tumor diameter from (a) 53 mm at the initial diagnosis to (b) 33 mm before cryoablation.

A 72-year-old man with a horseshoe kidney underwent heminephrectomy 13 years ago for right-sided renal cancer. Subsequently, he developed recurrent pulmonary and adrenal metastases, which were managed through surgical excision. A 53 mm renal neoplasm was identified in the lower pole of the left kidney, which was diagnosed as clear cell renal cell carcinoma (ccRCC) based on the biopsy results. Due to the considerable size of the tumor, preoperative administration of axitinib was initiated. Although mild renal impairment was observed, the tumor size decreased to 33 mm, allowing the safe implementation of cryoablation (Figure 1). While no local recurrence was detected, osseous metastasis from the right renal carcinoma necessitated systemic therapy with nivolumab.

Case 2

**Figure 2:**
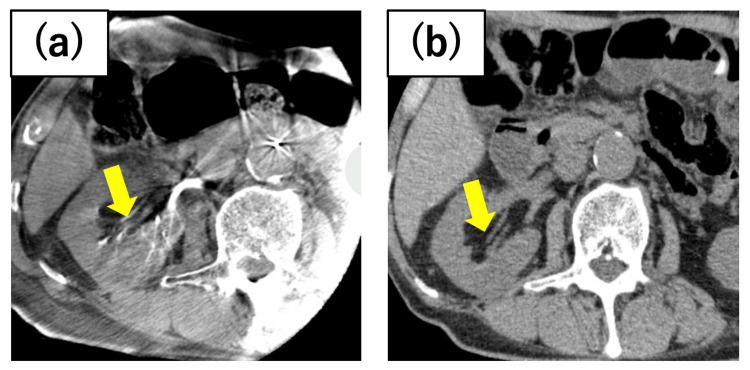
Case 2 renal tumor The tumor was located on the dorsal side of the right kidney, bordering the renal pelvis. Preoperative axitinib was administered, and the tumor shrank from (a) 41 mm in length at the initial diagnosis to (b) 27 mm before cryoablation.

An 82-year-old man presented with a 41 mm tumor in the middle of the right kidney. Ablative therapy was deemed appropriate given his medical history, which included recurrent peritonitis following surgery for gastric malignancy, atrial fibrillation, and renal dysfunction. To address concerns regarding adverse effects, axitinib was initiated at a dosage of 6 mg, and PCA was performed after tumor regression to 27 mm (Figure 2). No postoperative local recurrence was observed.

Case 3

**Figure 3:**
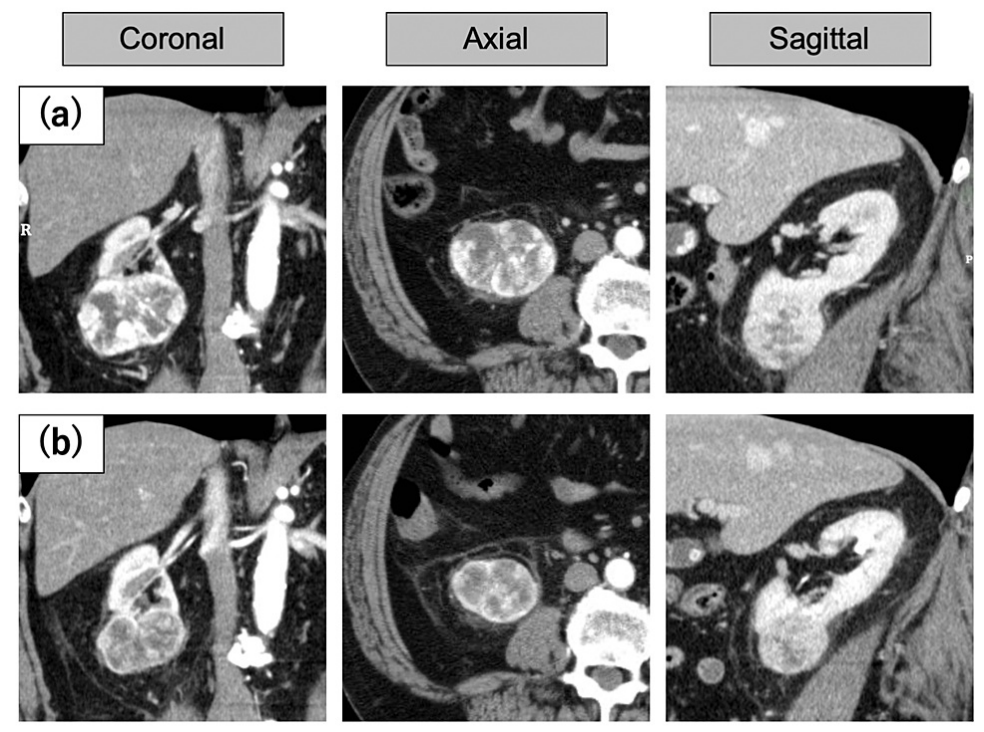
Case 3 renal tumor The contrast-enhanced CT of the renal tumor in Case 3 (a) shows the CT scan at the time of the initial diagnosis, revealing the tumor in direct contact with the iliopsoas muscle; (b) a CT scan before cryoablation after administration of molecular-targeted drugs. The tumor has undergone a reduction in both size and distance from the surrounding organs.

A 68-year-old man exhibited a 70 mm renal neoplasm in the lower pole of the right kidney. Due to the patient’s chronic obstructive pulmonary disease, rendering general anesthesia challenging, the approach of post-tumor reduction PCA was adopted. Axitinib was administered at a dosage of 10 mg; however, the patient developed proteinuria, prompting a switch to sunitinib after one month. Without experiencing any adverse effects, the tumor size decreased to 54 mm two months after the initiation of treatment (Figure 3). The tumor initially had extensive contact with the urinary tract, but due to tumor shrinkage, it only made partial contact with the renal calyx. As no further changes were observed four months after commencing treatment, cryoablation was performed. To date, there has been no local recurrence in this case.

Case 4

**Figure 4:**
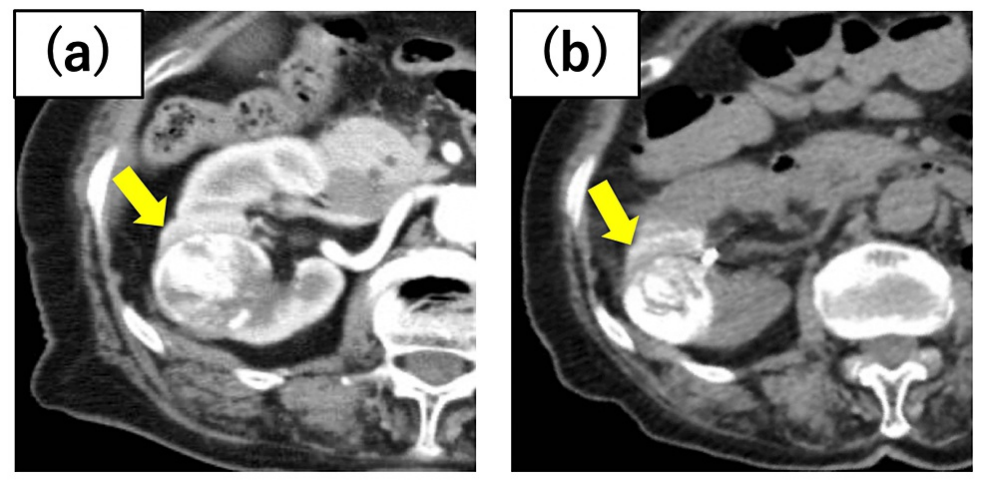
Case 4 renal tumor There were two tumors in the right kidney, and cryoablation was performed on the central tumor. Preoperative axitinib administration shrank the tumor diameter from (a) 41 mm at the initial diagnosis to (b) 29 mm before cryoablation.

A 79-year-old female patient had a solitary kidney following nephrectomy for left renal cell carcinoma. The imaging analysis revealed a 22 mm tumor in the upper pole of the right kidney and a 41 mm tumor in the central region. The central tumor was initially managed with axitinib, followed by PCA (Figure 4). Although the upper pole tumor exhibited gradual enlargement, no recurrence was observed at the ablated site.

Case 5

**Figure 5:**
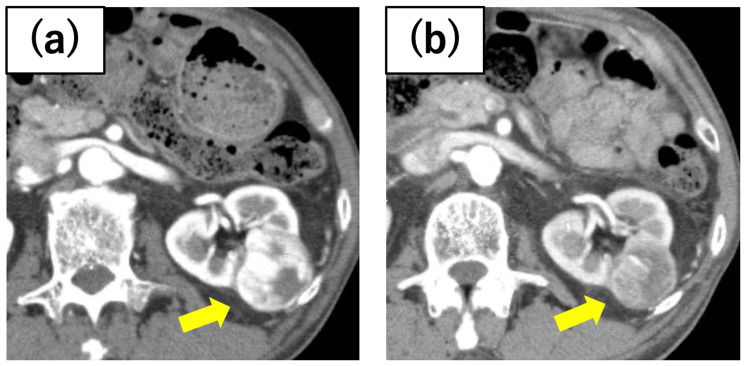
Case 5 renal tumor Preoperative axitinib administration resulted in tumor shrinkage from (a) 46 mm in tumor diameter at the initial diagnosis to (b) 39 mm before cryoablation. In this case, the reduction in contrast enhancement was particularly remarkable.

A 75-year-old man was referred to our institution with bilateral renal tumors. Due to hematopoietic insufficiency resulting from myelodysplastic syndrome, surgical intervention posed challenges, so PCA was considered appropriate. Preoperative treatment with axitinib was administered for a 46 mm tumor in the lower pole of the left kidney, resulting in tumor regression to 39 mm prior to PCA (Figure 5). While no local recurrence was evident on imaging, hematogenous metastasis to the contralateral retroperitoneum and bone was detected. Currently, the patient is engaged in discussions regarding the potential initiation of systemic treatment.

Case 6

**Figure 6:**
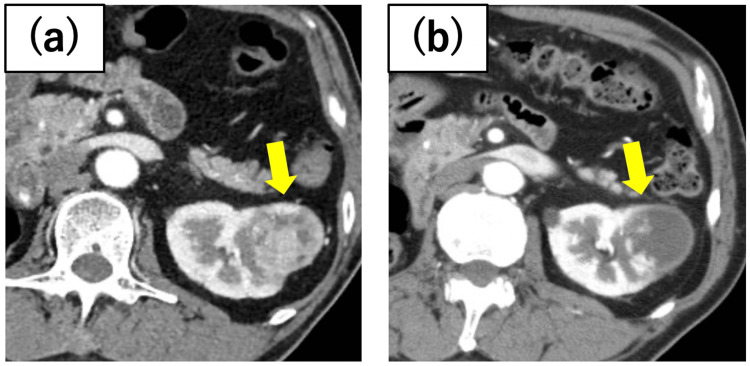
Case 6 renal tumor This is a case of a left solitary kidney. Preoperative axitinib administration resulted in a mild shrinkage in the tumor diameter from (a) 42 mm at the initial diagnosis to (b) 38 mm before cryoablation. However, there was a marked decrease in contrast enhancement.

A 67-year-old male patient with a solitary kidney following nephrectomy for right renal carcinoma 28 years ago presented with a 42 mm tumor in the upper pole of the left kidney and a 14 mm tumor in the lower pole. Axitinib for the upper pole tumor led to a decrease in contrast enhancement and tumor regression (Figure 6). PCA was performed without significant adverse effects for the upper pole tumor, and the lower pole tumor is under active surveillance. To date, no local recurrence has been observed, and the lower pole tumor has shown no changes.

## Discussion

There is substantial evidence that PCA is an appropriate curative intervention for renal cell carcinoma, particularly in cases where surgical resection presents challenges, such as in patients with renal function decline or in whom general anesthesia is contraindicated. The curative and safety aspects of PCA for T1a tumors have been extensively debated, with lower local control rates compared to initial treatments; however, the ability to perform retreatment in cases of recurrence has resulted in favorable tumor control. Furthermore, PCA shows favorable outcomes in terms of shortened hospitalization duration and other tumor-related outcomes [8].

Recent advances in surgical techniques, such as the widespread adoption of robotic surgery, have facilitated nephron-sparing treatments even for T1b or T2a lesions. In the domain of ablative therapies, such as PCA, techniques including hydrodissection and pneumodissection have enabled the treatment of tumors larger than T1a [9]. However, the position and size of the tumor have become crucial factors dictating the treatment limits due to the potential impact of PCA on adjacent organs and the urinary tract.

To date, only one case of effective molecular-targeted drug administration prior to local ablative therapy has been reported. In that report, preoperative TAE failed to achieve tumor shrinkage for a T1b tumor, but substantial tumor reduction and a complete response were observed following the administration of sunitinib, facilitating successful PCA.

There are two potential advantages to using molecular-targeted drugs prior to PCA for renal cancer. First, tumor shrinkage increases the physical distance between the tumor and adjacent organs, such as the intestine, pancreas, and liver, potentially minimizing adverse effects on these organs from freezing. In Case 3 presented here, the tumor was initially in direct contact with the renal pelvis, but drug administration resulted in only partial contact with the renal calyx, enabling the treatment to proceed without urinary tract injury. In addition, tumor shrinkage may reduce the number of cryoneedle insertions, potentially alleviating the burden on patients.

The second advantage is the expected decrease in tumor blood flow. Cases where molecular-targeted drugs were used exhibited decreased contrast enhancement in CT imaging, suggesting a reduction in tumor blood flow. Decreased tumor blood flow has been shown to lead to a decrease in hemorrhage and to enhance the effectiveness of PCA, particularly in cases where preoperative TAE is not feasible due to impaired renal function [10-12]. Therefore, the administration of molecular-targeted drugs may offer even greater benefits in such cases.

This study had several limitations, including the absence of histopathological diagnosis through biopsy in all cases. Typically, we perform a simultaneous biopsy with PCA if the tumor clearly exhibits characteristics of ccRCC. However, for tumors with atypical appearances in images, we conduct prior biopsies to determine the treatment strategy. Nevertheless, the diagnostic accuracy of imaging for ccRCC is high, with a median sensitivity and specificity of 88% and 75% and 87.5% and 89% for MRI [13]. Given that tumor shrinkage with drugs was observed in all cases presented here, this is unlikely to be a significant issue [14].

Another limitation was the utilization of axitinib as the initial medication, as this drug is not generally recommended as the first-line treatment for ccRCC. However, in a previous study, axitinib resulted in a partial response rate of 57.8% [15]. Moreover, preoperative axitinib has been found to be safe and effective for ccRCC with tumor thrombus [16]. Therefore, axitinib seems to be an effective cytoreductive drug. Furthermore, the wide range of dose adjustments and the relative ease of adjustment make it suitable for patients with such comorbidities.

## Conclusions

This study demonstrated favorable treatment outcomes, including tumor shrinkage, achieved through the safe use of molecular-targeted drugs prior to PCA. Compared to immunotherapy, molecular-targeted agents have more reversible side effects, allowing for safer administration. Systemic treatment with molecular-targeted agents for patients undergoing PCA, who often have comorbidities, raises concerns regarding potential adverse effects. While the target population may be limited, considering the significant benefits outweighing the risks, it may be worth considering this approach, particularly for larger tumors classified as T1b or above.
